# Improving Access to WHO Formulations of Alcohol-Based Hand Rub in Healthcare Facilities: A District-Wide Approach

**DOI:** 10.4269/ajtmh.22-0554

**Published:** 2023-05-15

**Authors:** Fred Tusabe, Judith Nanyondo, Matthew J. Lozier, Maureen Kesande, Olive Tumuhairwe, Martin Watsisi, Fred Twinomugisha, Alexandra Medley, Julius Mutoro, Mohammed Lamorde, David Berendes

**Affiliations:** ^1^ Infectious Diseases Institute, Makerere University, Kampala, Uganda;; ^2^ Division of Foodborne, Waterborne, and Environmental Diseases, Centers for Disease Control and Prevention, Atlanta, Georgia;; ^3^ Kabarole District Health Office, Kabarole District Local Government, Fort Portal, Uganda;; ^4^ International Water & Sanitation Centre – WASH, Fort Portal, Uganda;; ^5^ Makerere University School of Public Health, Kampala, Uganda;; ^6^ Kasese District Health Office, Kasese, Uganda

## Abstract

Alcohol-based hand rub (ABHR) is an effective hand hygiene measure to mitigate and prevent infectious disease transmission in healthcare facilities (HCFs); however, availability and affordability in low- and middle-income countries are limited. We sought to establish centralized local production of ABHR using a district-wide approach to increase provider access at all public HCFs in Kabarole and Kasese Districts in Western Uganda. Partner organizations worked with district governments to adapt and implement the WHO protocol for local ABHR production at the district scale. These groups identified and upgraded sites for ABHR production and storage to ensure recommended security, ventilation, and air conditioning. District governments selected technicians for training on ABHR production. Raw materials were sourced within Uganda. Alcohol-based hand rub underwent internal quality control by the production officer and external quality control (EQC) by a trained district health inspector before distribution to HCFs. We assessed ABHR production and demand from March 2019 to December 2020. All ABHR batches (*N* = 316) met protocol standards (alcohol concentration: 75.0–85.0%) with a mean of 79.9% (range: 78.5–80.5%). Internal quality control measurements (mean alcohol concentration: 80.0%, range: 79.5–81.0%) matched EQC measurements (mean: 79.8%, range: 78.0–80.0%). Production units supplied ABHR to 127 HCFs in Kasese District (100%) and 31 HCFs in Kabarole District (56%); 94% of HCFs were small (dispensary or next higher level). This district-wide production met quality standards and supplied ABHR to many HCFs where facility-level production would be unfeasible. Low- and middle-income countries may consider district models to expand ABHR production and supply to smaller HCFs.

## INTRODUCTION

Hand hygiene is a core infection prevention and control (IPC) method for preventing healthcare-associated infections (HAIs).^1^ Healthcare-associated infections pose a great burden to patient safety and the entire healthcare system globally.^2^ Healthcare workers’ hands can transfer pathogens that cause HAIs, such as *Staphylococcus aureus*, *Proteus mirabilis*, *Candida auris*,* Burkholderia cepacia*,* Pseudomonas aeruginosa*,* Klebsiella* spp., *Enterococcus* spp.,* Bacillus* spp.,* Acinetobacter baumannii* and other *Acinetobacter* spp., human parainfluenza virus 3, rhinovirus 14, and severe acute respiratory syndrome coronavirus-2 (SARS CoV-2).^3^^–^^5^

In response to the coronavirus disease 2019 (COVID-19) pandemic, various non-pharmaceutical public health measures were newly established or enhanced to prevent transmission, including the use of face masks and increased access to hand hygiene, both in healthcare facilities (HCFs) and communities.^6^^,^^7^ Alcohol-based hand rub (ABHR) and handwashing with soap and water are both effective hand hygiene methods for healthcare workers. The WHO promotes ABHR use in HCFs because of its fast-acting and broad-spectrum microbicidal activity with minimal risk of generating resistance to antimicrobial agents. Furthermore, ABHR is suitable for use in resource-limited or remote areas with a lack of sinks or other facilities for hand hygiene, among other factors.^2^ The WHO and the CDC recommend using ABHR that contains at least 60% alcohol at patient care points as a standard of patient care to reduce transmission of emerging and re-emerging infectious diseases.^8^^,^^9^ When hands are not visibly soiled, ABHR is effective at reducing the number of viable pathogens that cause many enteric diseases, viral hemorrhagic fevers, and respiratory illnesses, among others.^10^ Critically, SARS-CoV-2—the virus that causes COVID-19—is also highly susceptible to ABHR, with log reductions greater than 5.9 in laboratory settings.^11^^–^^13^

Access to ABHR in low- and middle-income countries (LMICs) is challenging because of costs and logistical barriers to obtaining commercially produced ABHR, leading to a need for local production. Nationally representative surveys of hospitals in selected low-income countries before the COVID-19 pandemic suggest that ≤ 56% of hospitals had access to ABHR.^14^ In Uganda, 1 L of commercial ABHR cost about $10 before the COVID-19 pandemic and increased to as high as $46/L during the peak of the pandemic.^15^ The WHO has published protocols for local, facility-level production of two ABHR formulations: one using ethanol and the other using isopropanol.^16^ WHO formulations are effective against all bacteria as well as enveloped and most non-enveloped viruses of public health relevance.^17^^,^^18^ Studies on the production of ABHR, generally at single HCFs in LMICs, including in Costa Rica, Italy, Mali, Kenya, Pakistan, Uganda, Rwanda, and Saudi Arabia, among others, have shown improvements in hand hygiene compliance and reduction of infection transmission.^8^^,^^19^^–^^21^ However, onsite ABHR production may not be cost-effective for all HCFs, particularly small, primary HCFs that lack appropriate space and the right cadre to support ABHR production yet are staffed by healthcare workers at the frontline of outbreaks,^19^^,^^22^^–^^24^ thereby hindering the universal health coverage strategy.^25^ Several attempts at commercial or partner-led centralized, local production of ABHR in countries like Uganda, Chad, and Sierra Leone have shown commercial production to be expensive and centralized local production to be non-sustainable.^21^^,^^26^^,^^27^

Given these challenges, there is a need to establish, pilot, and evaluate models of local ABHR production at scales beyond single facilities (e.g., regional or district-level production) that is led by district or local governments—with partner support—to ensure sustainable production and access for the smallest, frontline HCFs. To address this gap, the Infectious Diseases Institute at Makerere University (IDI), with the support of the CDC and the International Water and Sanitation Center, partnered with the local government to implement and evaluate local production of ABHR at a single centralized HCF for district-scale distribution in two districts in Uganda. Results from this project can inform guidelines to expand local ABHR production to a district or regional level in Uganda as well as other LMICs in a manner that engages local governments and best utilizes existing systems to support sustainability.

## MATERIALS AND METHODS

### Study population.

**Figure 1.**
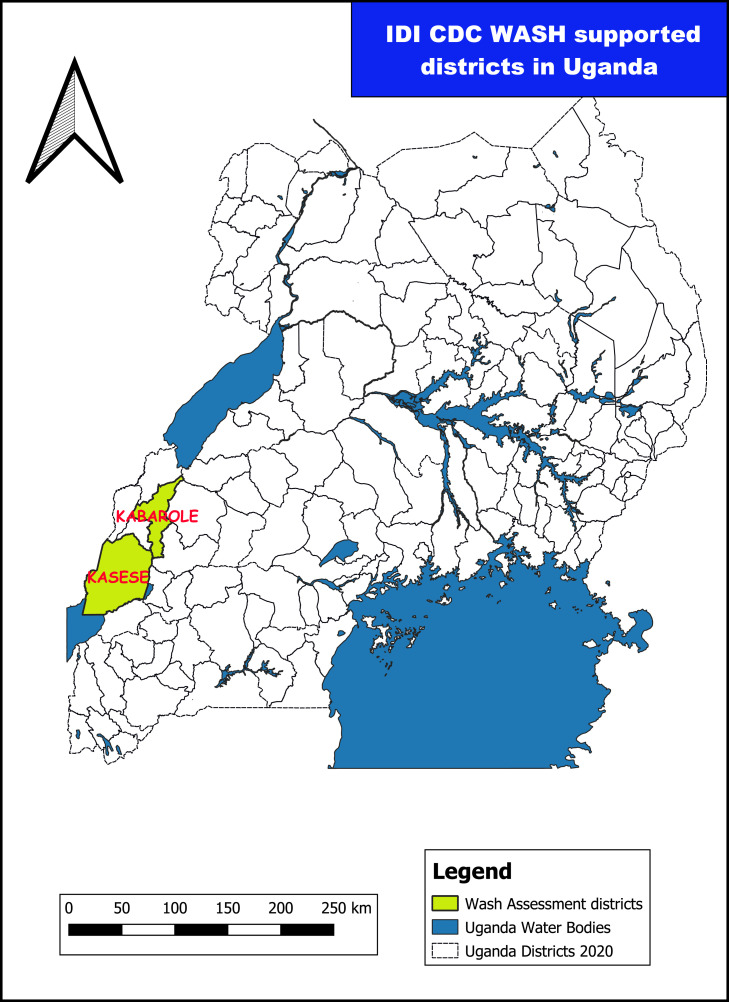
Map of Uganda showing districts with alcohol-based hand rub production units. IDI = Infectious Diseases Institute at Makerere University.

The Kabarole and Kasese Districts (Figure 1) are located in Western Uganda near the border of the Democratic Republic of Congo. Kabarole covers 1,814 km^2^. It has an estimated population of 325,000 and comprises 55 HCFs (31 public and 24 private).^28^ Kasese District covers 3,390 km^2^. It has an estimated population of 702,029 and comprises 127 HCFs (105 public and 22 private).^29^

### Identifying and targeting HCFs for the intervention.

The first year of implementation (January 2019–January 2020) was an efficacy trial of local ABHR production at a centralized location with distribution to all 30 primary public health centers (HCs) in Kabarole (phase 1: Kabarole). Health center level II (HC II) represents the smallest, dispensary-level, outpatient-only facilities; HC III represents medium-sized facilities with some inpatient care; HC IV represents facilities that are just smaller than a hospital, with multiple wards and surgical theaters, and hospitals were usually referral centers at a district or regional level. Following that period, a 1-year sustainability phase (January–December 2020) added a public hospital to make a total of 31 public health facilities (phase 1a: Kabarole). After cases of Ebola spilled over from the Democratic Republic of the Congo to Kasese District, Uganda, in June 2019,^30^ the ABHR intervention was expanded to include an additional production unit and support for ABHR for all (127) public and private HCFs in Kasese in December 2019–December 2020 (phase 2: Kasese).

### Identification and establishment of ABHR production units.

Alcohol-based hand rub production was established in January 2019 in Kabarole (for phase 1 and 1a) and in December 2019 in Kasese District (for phase 2) in Western Uganda (Figure 1). Both production units were selected to be centrally located in the districts, close to the district medical stores for coordination with existing district/national medical supply chains (e.g., National Medical Stores [NMS]) and ease of monitoring production.

### Local production of ABHR.

Production was conducted using standard operating procedures for ABHR production, safety, and quality control per the WHO protocol.^16^ The complete process is documented and described in Supplemental material 1 adapted from the WHO protocol. Trained staff spent approximately 40 minutes producing a 20-L batch of ABHR prior to quarantine, including packing and branding. This time reduced to about 20 minutes as the staff gained more proficiency.

### Quality assurance.

**Figure 2.**
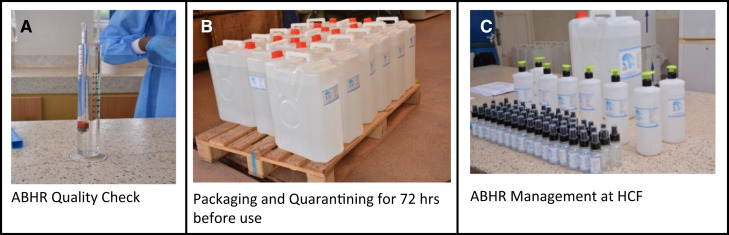
Alcohol-based hand rub (ABHR) quality check, packaging, and management at healthcare facilities (HCFs). (**A**) ABHR quality check. (**B**) Packaging and quarantining for 72 hours before use. (**C**) ABHR management at HCFs.

Alcohol-based hand rub alcohol content was assessed using an Economy Tralle & Proof alcoholmeter model number MT307 (Brewmaster, Pittsburg, CA) (Figure 2A). Internal quality control (IQC) consisted of measuring the alcohol concentration just after production to ensure that the ABHR met the WHO guidelines for ethanol-based ABHR (75.0–85.0%) (Figure 2A). Internal quality control was conducted by the ABHR production staff, and then the ABHR was put in quarantine for 72 hours to destroy any possible contaminating spores, per WHO guidelines for local production of ABHR^16^ (Figure 2B). External quality control (EQC) was conducted by district staff (district health inspectors [DHIs]) after quarantine but before distribution to ensure that the ABHR had maintained adequate alcohol concentration.

### Spot checks on ABHR alcohol concentration at HCF.

During the evaluation of the district model for ABHR production and distribution in Kabarole District (phase 1), ABHR was distributed to HCFs, and IDI conducted follow-up spot checks on the alcohol content of the ABHR using an alcoholmeter in HCFs that had retained the originally distributed ABHR for at least 12 months.

Data were analyzed using the statistical package IBM SPSS Statistics for Windows, version 22.0 software (IBM Corp., Armonk, NY).

### Training of district staff.

District health offices identified district staff, generally targeting laboratory technicians and pharmacists, to undergo a 3-day training on ABHR production facilitated by an experienced IDI officer using the WHO protocol.^16^ Both districts had few pharmacists; thus, laboratory technicians were trained to produce ABHR and conduct IQC. District health inspectors were selected and trained to conduct EQC.

### Sourcing of raw materials for production.

Hydrogen peroxide and sterile deionized water were procured from the NMS; ethanol was procured from Sugar Cooperation of Uganda Limited; and glycerol, mixing paddles, jars, alcoholmeters, mixing tanks, cylinders, and air conditioning systems were sourced from Transcell Biotech, one of the suppliers pre-qualified by IDI.

### Distribution of ABHR and management at HCFs.

During phase 1 in Kabarole District, distribution was done by the IDI team using hired vehicles (rather than via the NMS distribution system, which uses trucks to deliver essential medications quarterly to public HCFs). During phase 1a in Kabarole District, the district provided distribution via NMS vehicles, partner vehicles, or other in-kind methods. Similarly, during phase 2 in Kasese District, the initial distribution of ABHR (January 2020) was conducted using hired vehicles, but subsequent distribution was via the NMS distribution system. Periodically, some HCFs made direct requisitions to their respective District Health Office (DHO) and, upon approval, picked up the ABHR from the district stores. Otherwise, the delivery of ABHR was coordinated by the IPC focal person at the HCFs.

On delivery of the first batch of ABHR to HCFs, the IPC focal person and medical stores staff were trained on the management of ABHR stocks. Alcohol-based hand rub was distributed to HCFs in 20-L jerrycans, as shown in Figure 2B. This quantity was deemed sufficient to last multiple months at the smaller HCFs. The IPC focal person aliquoted the initial 20-L jerrycan into enough sterile 1-L pump bottles (Figure 2C) for each patient care point.^8^ Ugandan HCF stock cards were adapted for monitoring ABHR distribution within the HCFs from the jerrycan to points of care (Supplemental material 2). Healthcare workers were trained on when and how to use the ABHR utilizing the WHO’s “5 Moments of Hand Hygiene” and “How to Hand Rub Technique” job aids.^2^ Alcohol-based hand rub jerrycans were stored in medical stores at HCF, which were cool rooms. When 1-L pump bottles ran out of ABHR, staff at points of care were instructed to request more ABHR from the HCF medical stores, where the jerrycan with ABHR was stored. This request was documented on the Ugandan HCF stock card when the 1-L bottle was refilled. The IPC focal person was instructed to contact the DHO when the ABHR in the jerrycan reached 5 L to request additional ABHR delivery. The roles and responsibilities of entities within the ABHR district-led approach are outlined in Table 1.

**Table 1:** Roles and responsibilities of partners in ABHR district-wide approach for low- and middle-income countries

District health office	Implementing partners	District stores/national medical stores
Provide space for ABHR production unit and storage Identify district staff to be trained in ABHR production Produce ABHR, packaging, and branding Conduct internal quality control DHT conducts external quality control Manage ABHR supplies Strengthen ABHR use through mentorship programs in HCF	Supply needs and purchase of raw materials Establish and functionalize production unit including standard operating procedures Train district staff on ABHR production Technical support supervision Monitor consumption at HCFs Engage Ministry of Health for provision of supplies through NMS to enhance sustainability	Store ABHR raw materials Monitor stock levels Store final ABHR product and manage issuance to HCFs Support distribution of ABHR to HCF using NMS distribution networks

ABHR = alcohol-based hand rub; DHT = district health team; HCF = healthcare facility; NMS = national medical stores.

We initially distributed 20 L of ABHR to each HCF in Kabarole in February 2019 and to each HCF in Kasese in January 2020; we then refilled ABHR (i.e., provided more 20-L jerrycans) based on requests from the IPC focal persons at HCFs. We calculated consumption for phases 1 and 1a in Kabarole District and phase 2 in Kasese.

## RESULTS

From the establishment of the Kabarole ABHR production unit (February 2019) and the Kasese ABHR production unit (December 2019–December 2020), the Kabarole unit produced 5,420 L of ABHR, whereas the Kasese unit produced 9,980 L (Table 2). In both districts, the DHOs (local government) provided space for ABHR production and storage units. These units were existing structures assessed by a safety officer using a checklist adapted from WHO protocols (Supplemental material 3).^16^ In Kabarole, the room selected was in a HC IV. The room was well ventilated, cool, securable, away from sources of fire, and had clear walkways but lacked electricity and locks and had dirty paint. Air conditioning was determined not to be necessary for Kabarole District because of mild ambient temperatures. In Kasese, the room selected was at the DHO. The room was spacious, well ventilated, away from sources of fire, had clear walkways, and had electricity but lacked an air conditioning system, which was necessary due to the hotter climate.

**Table 2:** ABHR production and distribution costs

District	Item	2019(1)	2020(1a)	Total
Kabarole (phase 1 and 1a)	ABHR produced (L)	1,520	3,900	5,420
	Cost of raw materials (USD)	2,500	8,305	10,805
	Cost of one-time supplies* (USD)	4,817	0	4,817
	Cost of staff salaries† (USD)	13,597	13,597	27,194
	Overhead costs‡ (USD)	342	342	684
	Distribution costs§ (USD)	5,500	0	5,500
	Cost per liter (USD)	17.60	5.70	9.04
	Cost per ABHR liter (USD) without salaries, overhead and distribution costs (recurring cost)	4.81	2.13	2.88
Kasese (phase 2)	ABHR produced (L)	N/A	9,980	9,980
	Cost of raw materials (USD)	N/A	24,340	24,340
	Cost of one-time supplies* (USD)	N/A	9,041	9,041
	Cost of staff salaries† (USD)	N/A	0	0
	Overhead costs‡ (USD)	N/A	2,400	2,400
	Distribution costs§ (USD)	N/A	7,000	7,000
	Cost per liter (USD)	N/A	4.29	4.29
	Cost per ABHR liter (USD) without salaries, overhead and distribution costs (recurring cost)	N/A	3.34	3.34

ABHR = alcohol-based hand rub.

* One-off supplies included graduated cylinders, mixing tanks/buckets, alcoholmeters, air conditioning system, working benches, chairs, refurbishing of facilities, and personal protective equipment in Kasese and graduated cylinders, mixing tanks/buckets, alcoholmeters, workbenches, chairs, refurbishing of facilities, and personal protective equipment in Kabarole.

† In Kabarole, a study nurse/coordinator and ABHR producer was hired, who subsequently trained district staff whose time was donated in-kind. In Kasese, ABHR producers were assigned by the District Health Office, and their salary time was donated in-kind to the project.

‡ Overhead costs in Kabarole included stationary and ABHR production unit maintenance. In Kasese these costs were stationery, ABHR production unit maintenance, transport and lunch allowance to district producers, transportation, and accommodation for project staff who provided quarterly technical support to Kasese.

§ Distribution was paid by the project in phase 1 (Kabarole) and for the initial distribution of phase 2 (Kasese). Subsequently, distribution for phase 1a (Kabarole) and the remainder of phase 2 (Kasese) was through the National Medical Stores system, partner vehicles, or other in-kind methods and therefore did not incur additional project costs.

### Staff training.

Four laboratory technicians, two from each DHO, were trained to produce ABHR and conduct IQC. Two DHIs—one in Kabarole and the other in Kasese—were trained to perform the EQC of ABHR.

### Raw material and ABHR costs.

One-time costs of supplies, such as graduated cylinders, an alcoholmeter, an air conditioning system, and other expenses like workbenches, refurbishing of facilities and personal protective equipment, were approximately $4,817 for Kabarole and $9,041 for Kasese (Table 2). Recurring costs such as raw materials, overhead, distribution, and salaries were approximately $45,000 for Kabarole and $34,000 for Kasese. During 2019 in phase 1, the average cost of ABHR in Kabarole was $17.60/L with all costs—including one-time costs—included ($4.81/L without salaries, overhead, and distribution). In 2020 (phase 1a), as more ABHR was produced, one-time costs were eliminated, and distribution was transferred to NMS or other partner systems, the cost of ABHR reduced to $5.70/L ($2.13/L without salaries, overhead, and distribution). Over phases 1 and 1a, when staff salaries, overhead, and distribution costs were removed, the cost was reduced to $2.88/L. In Kasese, staff were not hired, and the cost of ABHR in 2020 was $4.29/L ($3.34/L when overhead and distribution costs were excluded). One-time supplies in Kasese ($9,041) accounted for $0.90 of each liter produced in 2020.

### Alcohol concentration: internal and external quality control and spot checks at HCFs.

All ABHR batches (*N* = 316) met WHO protocol standards, with a mean alcohol concentration of 79.9% (range: 78.0–81.0%) (Table 3). Internal quality control test results closely matched EQC results from all 316 batches produced. Mean alcohol concentration was 80.0% at IQC (range: 79.5–81.0%) and 79.8% at EQC (range: 78.0–80.0%). Detailed IQC and EQC results from all batches can be found in Supplemental material 4.

**Table 3:** ABHR internal and external quality control results

Assessment	*N*	Minimum	Maximum	Mean	SE	95% CI
Paired IQC-EQC*	316	78.0	81.0	79.9	0.023	79.81–79.91
IQC*	316	79.0	81.0	80.0	0.020	79.96–80.04
EQC*	316	78.0	80.0	79.8	0.028	79.72–79.83
Spot check†	7	77.0	80.0	79.0	0.378	78.72–79.28

ABHR = alcohol-based hand rub; EQC = external quality control; IQC = internal quality control; SE = standard error.

* IQC and EQC data from phases 1, 1a, and 2.

† Spot checks for alcohol concentration of ABHR at healthcare facilities was only conducted in seven healthcare facilities that had received locally produced ABHR during phase 1 in Kabarole and had retained the originally distributed ABHR for at least 12 months.

In the seven spot checks conducted in November 2020 in Kabarole, ABHR that had been in the HCF for about 20 months was found to have slightly lower concentrations of alcohol, with a mean alcohol concentration of 79.0% (range: 77.0–80.0%), which still met WHO optimal quality parameters (Supplemental material 5).

### ABHR distribution.

Production units supplied ABHR to 31 targeted HCFs in Kabarole District (out of 55 total) and all 127 HCFs in Kasese District. Of the 31 HCF in Kabarole, 12 were HC II, 16 were HC III, two were HC IV, and one was a regional referral hospital (the hospital was added to ABHR distribution for Phase 1a). During phase 1, 15 HCFs (5 HC IIs, 9 HC IIIs, 1 HC IV) were supplied during February–June 2019. The remaining 15 HCFs (7 HC IIs, 7 HC IIIs, 1 HC IV) were supplied during July–September 2019; during this time, the previously supplied HCFs received commercial ABHR provided by WHO for ebola response efforts. After October 2019 (end of phase 1, all of phase 1a), all 30 HCFs and an additional regional referral hospital were supplied with locally produced ABHR. Of the 127 HCFs in Kasese, 85 were HC II, 35 were HC III, 4 were HC IV, and 3 were hospitals.

**Figure 3.**
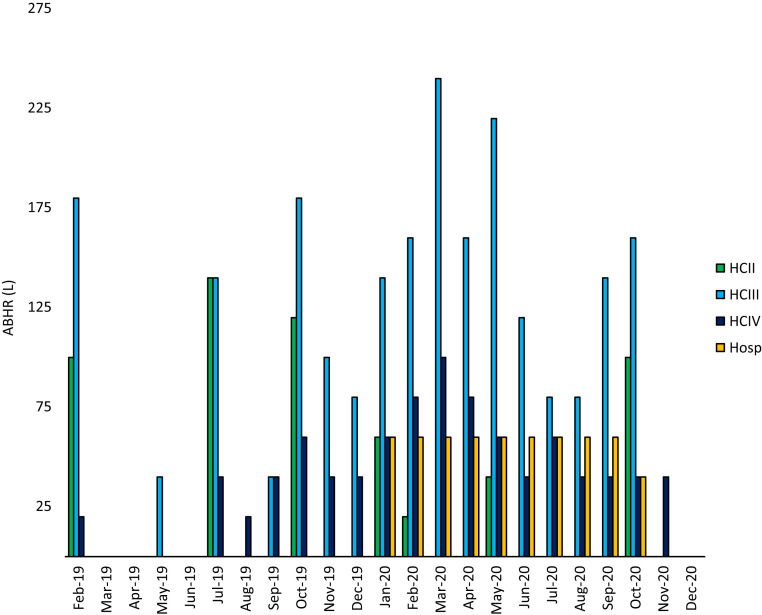
Total locally produced alcohol-based hand rub (ABHR) (L) distributed to healthcare facilities (HCFs) in Kabarole between February 2019 and December 2020. The number of HCFs receiving locally produced ABHR varied over time due to the ongoing efficacy trial and different phases of the project. From February to June 2019, 5 HC IIs, 9 HC IIIs, and 1 HC IV received ABHR that was locally produced (approximately half of the facilities due to the phase 1 design). Prior to the efficacy trial in late 2018, WHO distributed 10 L of commercially produced ABHR to all HCFs in response to Ebola outbreak concerns. From July to September 2019, 7 HC IIs, 7 HC IIIs, and 1 HC IV received locally produced ABHR (the other half of facilities due to the phase 1 design). In June 2019, the project team and local government worked to redistribute the remaining commercial ABHR to the 5 HC IIs, 9 HC IIIs, and 1 HC IV that previously received, locally produced ABHR; this redistribution ensured that ABHR—locally produced for those designated to receive it, or commercially made and previously distributed—was available for all HCFs in Kabarole District for the second half of 2019. From October 2019 to December 2020 (end of phase 1, all of phase 1a), 12 HC IIs, 16 HC IIIs, and 2 HC IVs received locally produced ABHR. One hospital received locally produced ABHR from January to December 2020.

A total of 5,420 L of locally produced ABHR were distributed to HCFs in Kabarole between February 2019 and December 2020 (Figure 3). In Kabarole, the largest monthly quantity of ABHR distributed was in March 2020, and the lowest was in December 2020.

**Figure 4.**
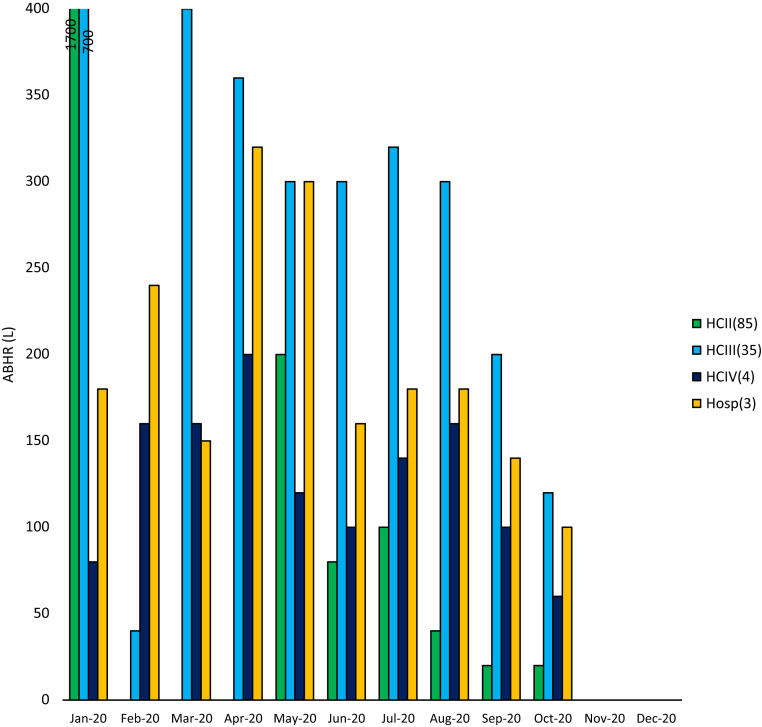
Total alcohol-based hand rub (ABHR) (L) distributed to healthcare facilities in Kasese District between January and December 2020 (phase 2).

A total of 9,980 L of ABHR were distributed to HCFs in Kasese (Figure 4), with the largest distribution in January 2020 and the lowest in December 2020.

### ABHR consumption.

The consumption rate for ABHR varied by HCF level (Table 4). In both districts, the highest ABHR consumption was in April 2020. The monthly mean consumption amounts of ABHR in HC II, III, IV, and hospitals in Kabarole District were 2.3 L, 7.2 L, 23.3 L, and 48.3 L, respectively, and 2.1 L, 6.9 L, 26.1 L, and 49.2 L in Kasese District, respectively.

**Table 4:** Monthly consumption of locally produced ABHR in liters

Month and year*	Kabarole: L ABHR consumed (no. of HCFs)					Kasese†: L ABHR consumed (no. of HCFs)				
	HC II	HC III	HC IV	Hospital	Total	HC II	HC III	HC IV	Hospital	Total
March 2019	7 (5)	38 (9)	0 (1)	–	45 (15)	–	–	–	–	–
April 2019	7 (5)	42 (9)	8 (1)	–	57 (15)	–	–	–	–	–
May 2019	12 (5)	49 (9)	4 (1)	–	65 (15)	–	–	–	–	–
June 2019	10 (5)	46 (9)	4 (1)	–	60 (15)	–	–	–	–	–
July 2019	16 (7)	62 (7)	28 (1)	–	106 (15)	–	–	–	–	–
August 2019	17 (7)	57 (7)	17 (1)	–	91 (15)	–	–	–	–	–
September 2019	20 (7)	65 (7)	28 (1)	–	113 (15)	–	–	–	–	–
October 2019	30 (12)	73 (16)	38 (2)	–	141 (30)	–	–	–	–	–
November 2019	28 (12)	68 (16)	42 (2)	–	138 (30)	–	–	–	–	–
December 2019	21 (12)	51 (16)	31 (2)	–	103 (30)	–	–	–	–	–
January 2020	16 (12)	64 (16)	35 (2)	60 (1)	175 (31)	90 (85)	188 (35)	32 (4)	90 (3)	400 (127)
February 2020	23 (12)	67 (16)	48 (2)	60 (1)	198 (31)	123 (85)	210 (35)	43 (4)	180 (3)	556 (127)
March 2020	22 (12)	153 (16)	60 (2)	60 (1)	295 (31)	178 (85)	315 (35)	120 (4)	150 (3)	763 (127)
April 2020	36 (12)	255 (16)	100 (2)	60 (1)	451 (31)	296 (85)	400 (35)	190 (4)	300 (3)	1.186 (127)
May 2020	30 (12)	230 (16)	80 (2)	60 (1)	400 (31)	200 (85)	340 (35)	185 (4)	280 (3)	1.005 (127)
June 2020	34 (12)	160 (16)	60 (2)	60 (1)	314 (31)	220 (85)	360 (35)	140 (4)	160 (3)	880 (127)
July 2020	30 (12)	160 (16)	60 (2)	60 (1)	310 (31)	170 (85)	310 (35)	130 (4)	180 (3)	790 (127)
August 2020	28 (12)	140 (16)	80 (2)	60 (1)	308 (31)	172 (85)	330 (35)	150 (4)	180 (3)	832 (127)
September 2020	32 (12)	140 (16)	60 (2)	60 (1)	292 (31)	200 (85)	260 (35)	110 (4)	150 (3)	720 (127)
October 2020	30 (12)	120 (16)	40 (2)	40 (1)	230 (31)	160 (85)	110 (35)	100 (4)	100 (3)	470 (127)
November 2020	28 (12)	80 (16)	29 (2)	0 (1)	137 (31)	150 (85)	70 (35)	55 (4)	0 (3)	275 (127)
December 2020	24 (12)	22 (16)	11 (2)	0 (1)	57 (31)	164 (85)	25 (35)	0 (4)	0 (3)	189 (127)
Total L ABHR (March 2019– December 2020)	501	2,142	863	580	4,086	2,123	2,918	1,255	1,770	8,066
Mean L ABHR/HCF/month‡	2.3	7.2	23.3	48.3	7.2	2.1	6.9	26.1	49.2	5.3

ABHR = alcohol-based hand rub; HC = health center; HCF = healthcare facility.

* During the study period (February–November 2019) most HCFs had other ABHR brands donated to them at a time Uganda was threatened by an Ebola outbreak. Consumption monitoring excluded the other brands; we only focused on our brand. In early 2020, there was primarily locally produced ABHR across all healthcare facilities, and during this time we initiated distribution to the only public hospital in Kabarole District.

† ABHR distribution in Kasese began January 2020.

‡ During months where HCFs were receiving/had previously received ABHR.

## DISCUSSION

This project demonstrated the successful implementation of a component of the WHO multimodal hand hygiene strategy^31^—local production of ABHR—for HCFs through government partnership at a district scale in a LMIC setting. Through these efforts, ABHR was provided to 158 HCFs across two districts in Uganda, providing access to a critical hand hygiene resource before and during the COVID-19 pandemic, and ABHR consumption was monitored, producing practical, facility-level estimates for future preparedness efforts. This implementation model is the first, to our knowledge, that is government led at the district level in Uganda and sub-Saharan Africa and holds promise for expansion to other similar contexts or scaling it to regional or national levels. In addition to Kasese and Kabarole, we are currently replicating this approach in six districts Uganda: Tororo and Amuru Districts (border districts), Kampala (capital), and Kotido and Moroto Districts (Karamoja Region) and Koboko (west Nile), with additional considerations for integration into emergency operations planning and response activities.

Although ABHR produced by the local production protocol does not have a pre-defined shelf life,^16^ all ABHR met IQC and EQC standards both at the production unit and up to 20 months after production at HCFs. This finding is comparable to ABHR samples tested in a tropical climate in Mali that still met protocol standards after 19 months without special air conditioning.^16^ However, targeted research on ABHR shelf-life from these protocols could improve planning for managing logistics and pre-positioned stockpiles in larger local production systems.

This model of ABHR production for district-level distribution demonstrated a partnership between the local government and implementing partner. District or other local governments can support local ABHR production in collaboration with local partners and enable all HCFs to have access to quality-controlled ABHR. The DHO identified and provided space for the production unit, which is near district-level medical supply stores to enable ease of distribution using the available district last-mile distribution systems, as well as portions of staff time for production and monitoring. Infectious Diseases Institute at Makerere University provided raw materials and technical support. Training DHO office staff to produce and monitor ABHR improved capacity and made the model more sustainable by reducing staffing costs.

Costs per liter of ABHR in Kabarole District during phase 1 were higher because of the staff hired for ABHR production and coordination. Although costs in phase 1 were $17.60/L in Kabarole District, about $9 of that cost was attributed to study staff for the efficacy trial, which was more intensive than regular, sustained production. In 2020 (phase 1a), even with study staff salaries, costs dropped to $5.70/L because there were no startup costs, and the DHO was able to eliminate distribution costs by leveraging the existing NMS distribution network and partner distribution systems. Although Kasese District (phase 2) had significant startup costs ($9,000) and initial distribution costs ($7,000), it effectively eliminated staff costs by training existing district health staff to produce ABHR. Kasese District also used NMS to distribute ABHR after initial distribution, which would make operational costs beyond this project even lower than the $4.29/L observed during phase 2. Once one-time costs are accounted for, recurring production costs drop to around $3/L in both districts. In Uganda, 1 L of commercial ABHR cost about $10 pre-pandemic and increased more than 4-fold during the peak of the pandemic.^15^ Within phases 1a and 2 of the study, ABHR costs were relatively low compared with the local commercial market. The lower cost of locally produced ABHR compared with commercially available ABHR may make it more affordable for healthcare systems to incorporate into the district, regional, or national budgets. Careful consideration of existing local expertise (e.g., laboratory technicians) and distribution networks could help ABHR program planners in other settings reduce costs.

We also identified consumption rates to inform needs by the level of HCF, which can help in planning for overall ABHR production needs and costs. This study monitored the consumption of the ABHR it provided during phase 1; however, commercial ABHR distributed by WHO/UNICEF to all HCFs in Kabarole District in late 2018 prior to phase I of our study likely means that consumption rates are underestimated during that period. Consumption rates may be more reliable during the latter phase of phase 1 (when the district government redistributed remaining commercial ABHR to HCFs not receiving local ABHR, June–September 2019) and during phases 1a and 2. Countries may consider the guidance provided by WHO on how much ABHR is needed per month to determine initial distribution amounts in their settings.^32^ Consumption of ABHR varied over time, likely due to mass media education on hand hygiene and risk perception at a time Uganda registered its COVID-19 disease index case, among other factors.^15^^,^^33^^,^^34^ Hand hygiene practices, assessed separately, were higher after ABHR production and distribution compared with before, indicating that this type of program can improve hand hygiene adherence among healthcare workers.^34^ However, overall hand hygiene rates were well below 100% compliance, which should be accounted for when evaluating ABHR consumption estimates.

Quality control of ABHR packaging is important to meet WHO standards and may also affect costs. The WHO formulations recommend that new ABHR dispensers be used with each distribution or that dispensers be sterilized between uses.^16^ Two-thirds of 39 ABHR production units in one evaluation reused dispenser bottles because it was more cost-effective and because it was sometimes difficult to procure ABHR packaging, but almost half of those had suboptimal disinfecting between uses.^19^ In this study, 1-L ABHR bottles were refilled from the 20-L jerrycan in the HCF store but were not disinfected before refill because of the limited availability of disinfection materials and protocols, especially in small facilities. Future versions of this protocol should include evaluations of field-feasible on-site sterilization methods and incorporate them into production costs.

### Considerations for replication.

After assessment of and approval for the physical sites to be used for ABHR production, each was converted into functional production units within about 7 working days. The time required to convert these sites into functional ABHR production units may vary depending on modifications needed and procurement processes in different districts, regions, or countries.

After local laboratory technicians were trained to produce ABHR, they were closely supervised by the IDI technical staff while producing their first lot of ABHR; subsequently, they produced ABHR independently with close supervision of the district IPC officer or DHI, whereas IDI continued to provide quarterly oversight. Although the exact time required for production by these technicians was not quantified and was donated in-kind by the DHO, production was not a full-time job because production of 20 L of ABHR takes approximately 20 minutes once staff are trained.^35^ At this rate, the total annual quantity produced for Kasese District in 2020 could be completed in fewer than 14 hours per month. Primarily, technical oversight from a partner with experience in ABHR production was the major need for initial setup and training, though not a continuous need. These needs, and associated roles and responsibilities, may change in other countries or when considering outbreak (versus the current, non-outbreak) contexts.

Infectious Diseases Institute at Makerere University sent the ABHR samples to the national regulatory authority—the National Drug Authority—where samples were tested and approved upon passing all the parameters, including alcohol content, according to the Ugandan standard (US EAS 789:2013); however, countries must identify and involve regulatory authorities to perform these quality checks and supervisions before mass production.

In this project, ethanol was used because isopropanol was not readily available; however, all ABHR raw materials were widely available in Uganda. Consideration of the availability of raw materials is critical in other LMICs; differences in alcohol type could be also subject to licensing restrictions.

### Sustainability benefits and challenges.

To date, this model is in multiple districts in Uganda, but there is a need for national adoption to ensure sustainable access in Ugandan healthcare facilities and community settings during both outbreak and non-outbreak periods. To support sustainability, the project developed a virtual curriculum on local production of ABHR, which can systematize training and reduce time required of technical experts. Alcohol-based hand rub production within this project has also supported preparedness and response to different disease outbreaks.^34^

However, challenges remain to develop a fully sustainable local production model. Within the described program, identifying reliable sources of certain raw materials (e.g., glycerol) from existing medical stores was difficult, and the transfer of costs to the district, regional, and national entities is still in progress. Additionally, sustainable distribution and monitoring of consumption—including identification of forms, roles and responsibilities, and transport—may require setting-specific solutions depending on what systems are already in place. The WHO guidance for local production provides standards for the safety of facility-level production, but guidelines for larger scales of production are needed to ensure that safe storage and production are possible. Finally, district-level production may not be the most sustainable option if countries have a national centralized capacity for production; however, countries may consider a combination of designs to include national production with regional/district-level surge capacity, such as capacity or pre-positioning of ABHR at emergency operations centers for outbreak response.

## CONCLUSION

Because local production of ABHR is not feasible on a facility level for HCFs of all sizes, centralized, district-led production of ABHR can provide a greater proportion of HCFs, regardless of size or capacity, with regular access to quality-assured ABHR. Low- and middle-income countries may consider district models to expand ABHR production using examples from the current program in Uganda to ensure production, quality control, distribution, and monitoring of consumption are successful and sustainable.

## Supplemental Materials


Supplemental materials

Supplemental materials

Supplemental materials
