# Supplementary material for: Improving Access to WHO Formulations of Alcohol-Based Hand Rub in Healthcare Facilities: A District-Wide Approach
Source: Am J Trop Med Hyg. 2023 May 15;109(1):191–200. doi: 10.4269/ajtmh.22-0554 (PMC10324005; doi:10.4269/ajtmh.22-0554)
Supplement: Supplementary file 1 [file tpmd220554.SD1.pdf]

Local Production of ABHR

Production was carried in production units with standard operating procedures (SOPs) for ABHR production, safety, and quality control. During production, personal protective equipment (PPE) was mandatory, including a mask, nitrile gloves, goggles, apron and boots. Batches of 20 litres each were produced. On average, it required 20 to 30 minutes to produce a batch.

REAGENTS FOR FORMULATION 1:

- Ethanol 96%
- Hydrogen peroxide 3 or 6%
- Glycerol 98-100%
- Sterile de-ionized or boiled cold water

REAGENTS FOR FORMULATION 2:

- Isopropyl alcohol 99.8%
- Hydrogen peroxide 3 or 6%
- Glycerol 98-100%
- Sterile de-ionized or boiled cold water

Reagents for production

Roles of each ingredient

- Hydrogen peroxide: Inactivates contaminating bacterial spores that may be in the solution and is not an active substance for the ABHR.
- Glycerol: used as emollients or humectant. Different emollients may be used for skin care, provided that they are cost-effective, miscible in water and alcohol, widely available and do not add to toxicity, or promote allergies.
- Sterile De-ionized water/ cold boiled water: Used as a universal solvent
- Ethanol/ Isopropyl alcohol: Active ingredient works by denaturing lipid and protein membranes of microorganisms (Coagulation)

Note:

1. Countries may consider adding perfumes, a colorant or dyes to allow differentiation from other products, but should not add to toxicity, promote allergy, or antagonize with antimicrobial properties.
2. Any extra additive to both formulations should be clearly labeled and be non-toxic in case of accidental ingestion
3. Addition of colorants, perfumes, dyes or extra additives increases costs of production.

Materials

- 20l plastic jerry can with a screw top,
- 50l plastic tank, wooden mixing paddle,
- 2000mls Measuring jar,
- 10mls,50ml, 100mls, 100mls 2000mls Measuring cylinders
- Working table/bench
- Waste bins
- Protective equipment i.e Goggles/Face shield, Face masks, Nitrile gloves, Gumboots, Aprons.
- Isopropyl or Ethanol alcoholmeter: the temperature scale is at the bottom and the ethanol /isopropyl concentration (percentage v/v) at the top.

METHOD: 20-LITRE PREPARATIONS OF ABHR

These quantities can be prepared in a 50-litre plastic tank to avoid overflowing.

Recommended amounts of products:

FORMULATION 1

- Ethanol 96%: 16667 ml

FORMULATION 2

- Isopropyl alcohol 99.8%: 15030 ml

- Hydrogen peroxide 3%: 833ml
- Glycerol 98-199%: 290ml
- Sterile de-ionized water:2210 ml

- Hydrogen peroxide 3%: 833 ml
- Glycerol 98-100%: 290 ml
- Sterile de-ionized water:3847 ml

Stepwise process for 20-litre preparation of ABHR

- In a 50l, graduated container, first, add 16667 ml of absolute ethanol (96%)
- Add pour in a clean bucket. 833 ml of 3% hydrogen peroxide measure and dispense 290 ml of glycerol making sure it all drains into the mixture.
- Top-up with de-ionised sterile water or cold boiled water up-to-the 20-litre mark alternatively directly measure 2210 ml of de-ionised sterile water into the mixture
- Using a mixing paddle, mix the solution gently back and forth
- Pack the product in a clean container/ 20 l jerry can.
- Label the container to include the date of production, batch number, ingredients used, Alcohol final concentration.
- At the production units, internal quality control is performed using an alcohol meter immediately after production. A concentration of (80±5%) ethanol content is acceptable for formulation 1 in figure (2A) and (75±5%) for formulation 2 is acceptable.
- Let the product sit for 72 hours before use to kill any contaminating spores. The product was stored at the district drug stores before distribution.
- Countries may also consider external quality control before dispatch to facilities.

WHO-Recommended Formulation

FORMULATION 1

Final concentrations:

- Ethanol 80% (v/v),
- Glycerol 1.45% (v/v),
- Hydrogen peroxide 0.125% (v/v)

FORMULATION2

Final concentrations:

- Isopropyl alcohol 75% (v/v),
- Glycerol 1.45% (v/v),
- Hydrogen peroxide 0.125% (v/v)

Available from: guide-to-local-production.pdf (who.int)[16]

HEALTH CARE STOCK CARD

(1) Facility Name:____ (2)Facility location:___________ _______

(3) Financial Year:____

Item Description (Name, Formulation, Strength):							Pack Size		Code No	
Special Storage Conditions										
Unit Of Issues				Maximum Stock Level			Minimum Stock Level			
Date	To Or From	Voucher Number	Qty in	Qty in	Losses/ Adjustments	Balance On Hand	Expiry Date	Batch No.	Remarks	Initials

Alcohol-Based Handrub Production & Storage Unit Selection Checklist

Inspection Team Members: ___

Inspection Date: ______________

Unit Room No/Location: ______

Instructions:

- Boxes to be marked ticked (√) as items are identified or with a cross(x) if not present. ***Any question marked with a cross requires corrective action.***
- Any corrective actions which cannot be immediately resolved are to be entered into the comments section to assess the risk and determine appropriate controls, responsibilities and time frames if appropriate.
- If the corrective action is immediately completed please write "Completed" in the comment section.
- If the space identified is not large enough to accommodate production and storage, request for the availability of a second room and asses the rooms as production and storage facilities independently.
- After completion of the checklist/s, email it/them to the project officer to make a final decision.

Criteria	YES	NO	Comment
Production and Storage Facility			
The general area is tidy and free of obstruction and mess.	☐	☐	
Security arrangements are in place and the area has restricted access	☐	☐	
The anticipated facility is lockable, secured and shielded as required.	☐	☐	
Security arrangements are in place and the area has restricted access	☐	☐	
Stair treads if available are in good condition (no worn or broken treads).	☐	☐	
Walkways/floor areas are clear of obstructions and trip hazards (e.g. electrical cords).	☐	☐	
All windows and doors are sealed and there are no gaps	☐	☐	
Temperature is comfortable (cool)	☐	☐	
The facility is well ventilated	☐	☐	
Facility has electricity	☐	☐	
Lighting adequate and operational	☐	☐	
The facility is away from naked flames/ sources of fire	☐	☐	
The facility is spacious to have separate work and storage area, administration tasks and associated paperwork.	☐	☐	
The facility has running water	☐	☐	

Criteria	YES	NO	Comment
Storage Facility			
The general area is tidy and free of obstruction and mess.	☐	☐	
The facility is close to the production Unit, if No there are means of transport to and from the facilities	☐	☐	
Anticipated storage sites are lockable, secured and shielded as required.	☐	☐	
Security arrangements are in place and the area has restricted access	☐	☐	
Stair treads are in good condition (no worn or broken treads).	☐	☐	
Walkways/floor areas are clear of obstructions and trip hazards (e.g. electrical cords).	☐	☐	
All windows and doors are sealed and there are no gaps	☐	☐	
Temperature is comfortable (cool)	☐	☐	
The facility is away from naked flames/ sources of fire	☐	☐	

Other comments
